# Poly[[diaquabis(2,2′-bipyridine-κ^2^ *N*,*N*′)(μ_3_-5-hydroxyisophthalato-κ^5^ *O* ^1^,*O* ^1′^:*O* ^3^,*O* ^3′^:*O* ^3′^)(μ_3_-5-hydroxy­isophthalato-κ^4^ *O* ^1^,*O* ^1′^:*O* ^3^:*O* ^3′^)(μ_2_-5-hydroxyisophthalato-κ^3^ *O* ^1^,*O* ^1′^:*O* ^3^)didysprosium(III)] dihydrate]

**DOI:** 10.1107/S1600536811039080

**Published:** 2011-09-30

**Authors:** Yan-Lin Zhang

**Affiliations:** a School of Chemistry and Environment, South China Normal University, Guangzhou 510006, People’s Republic of China

## Abstract

The polymeric title compound, {[Dy_2_(C_8_H_4_O_5_)_3_(C_10_H_8_N_2_)_2_(H_2_O)_2_]·2H_2_O}_*n*_, contains two independent Dy^III^ ions, both of which are nine-coordinated in a distorted tricapped trigonal–prismatic geometry. One Dy^III^ ion is coordinated by five 5-hy­droxy­isophthalate (hip) ligands and one 2,2′-bipyridine (bpy) ligand and the other by three hip ligands, one bpy ligand and two water mol­ecules. The Dy^III^ ions are bridged by the carboxyl­ate groups of the hip ligands, forming a three-dimensional framework. O—H⋯O hydrogen bonds are present in the crystal structure.

## Related literature

For related structures, see: Li *et al.* (2007 ▶); Plater *et al.* (2001 ▶); Zhuo *et al.* (2006*a* ▶,*b* ▶).
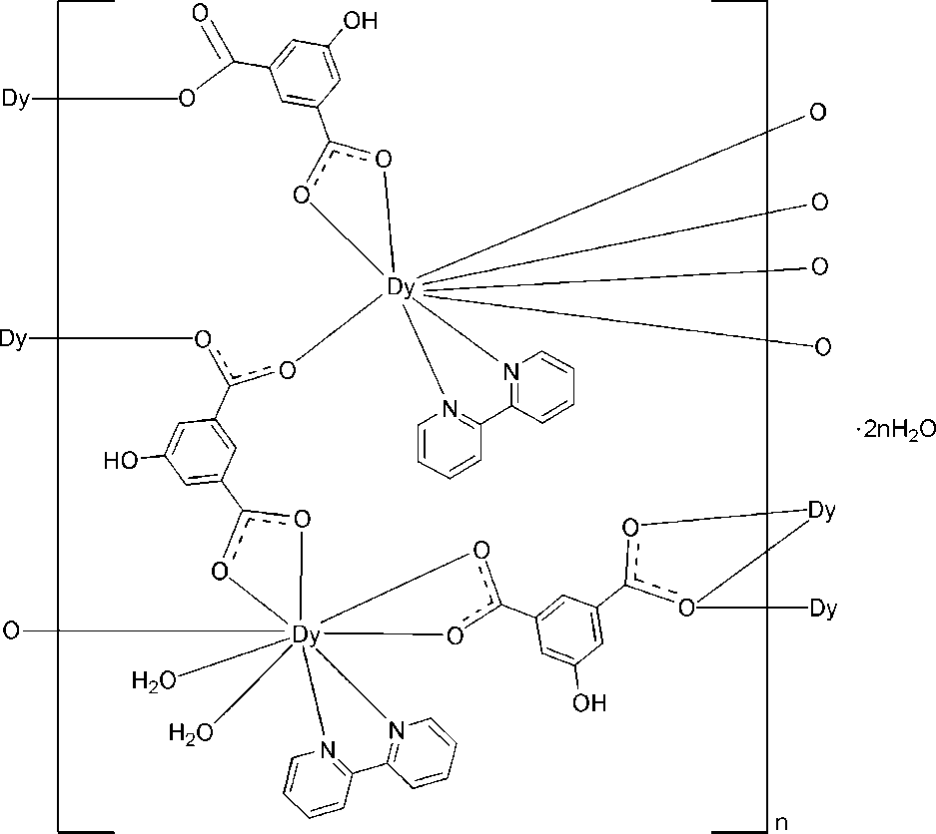


## Experimental

### 

#### Crystal data

[Dy_2_(C_8_H_4_O_5_)_3_(C_10_H_8_N_2_)_2_(H_2_O)_2_]·2H_2_O
*M* *_r_* = 1249.77
Triclinic, 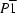

*a* = 11.3736 (5) Å
*b* = 12.0349 (6) Å
*c* = 17.8360 (8) Å
α = 91.310 (1)°
β = 103.187 (1)°
γ = 106.505 (1)°
*V* = 2269.00 (18) Å^3^
*Z* = 2
Mo *K*α radiation
μ = 3.35 mm^−1^
*T* = 298 K
0.25 × 0.24 × 0.21 mm

#### Data collection

Bruker APEXII CCD diffractometer
Absorption correction: multi-scan (*SADABS*; Bruker, 2001 ▶) *T*_min_ = 0.488, *T*_max_ = 0.540
11732 measured reflections
8024 independent reflections
7052 reflections with *I* > 2σ(*I*)
*R*_int_ = 0.020

#### Refinement

*R*[*F* ^2^ > 2σ(*F* ^2^)] = 0.035
*wR*(*F* ^2^) = 0.094
*S* = 1.06
8024 reflections
624 parameters
252 restraints
H-atom parameters constrained
Δρ_max_ = 2.65 e Å^−3^
Δρ_min_ = −1.25 e Å^−3^

### 

Data collection: *APEX2* (Bruker, 2007 ▶); cell refinement: *SAINT* (Bruker, 2007 ▶); data reduction: *SAINT*; program(s) used to solve structure: *SHELXS97* (Sheldrick, 2008 ▶); program(s) used to refine structure: *SHELXL97* (Sheldrick, 2008 ▶); molecular graphics: *DIAMOND* (Brandenburg, 1999 ▶) and *SHELXTL* (Sheldrick, 2008 ▶); software used to prepare material for publication: *SHELXTL*.

## Supplementary Material

Crystal structure: contains datablock(s) I, global. DOI: 10.1107/S1600536811039080/hy2467sup1.cif

Structure factors: contains datablock(s) I. DOI: 10.1107/S1600536811039080/hy2467Isup2.hkl

Additional supplementary materials:  crystallographic information; 3D view; checkCIF report

## Figures and Tables

**Table 1:** Hydrogen-bond geometry (Å, °)

*D*—H⋯*A*	*D*—H	H⋯*A*	*D*⋯*A*	*D*—H⋯*A*
O5—H5⋯O4*W*^i^	0.82	2.15	2.910 (11)	154
O10—H10*A*⋯O2^ii^	0.82	1.95	2.761 (6)	168
O15—H15⋯O1^iii^	0.82	1.92	2.718 (6)	163
O1*W*—H1*WA*⋯O8^iv^	0.85	1.84	2.609 (6)	150
O1*W*—H1*WB*⋯O3*W*^iii^	0.85	2.01	2.717 (7)	141
O2*W*—H2*WA*⋯O11	0.85	2.15	2.676 (6)	120
O2*W*—H2*WB*⋯O7^ii^	0.85	1.86	2.689 (6)	164
O3*W*—H3*WA*⋯O4*W*^iii^	0.85	1.99	2.744 (9)	147
O3*W*—H3*WB*⋯O8^v^	0.85	2.03	2.832 (9)	156
O4*W*—H4*WA*⋯O6^vi^	0.85	1.96	2.809 (8)	175
O4*W*—H4*WB*⋯O12	0.85	2.20	2.801 (8)	128

Symmetry codes: (i) 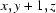; (ii) 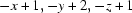; (iii) 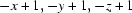; (iv) 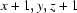; (v) 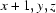; (vi) 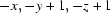.

**Fig. 1.**
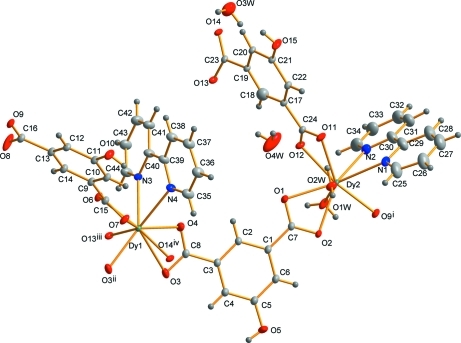
The asymmetric unit of the title compound. Displacement ellipsoids are drawn at the 30% probability level. [Symmetry codes: (i) x+1, y, 1+z; (ii) -x, 2-y, 1-z; (iii) -x, 1-y, 1-z; (iv) -x, 1+y, z.]

**Fig. 2.**
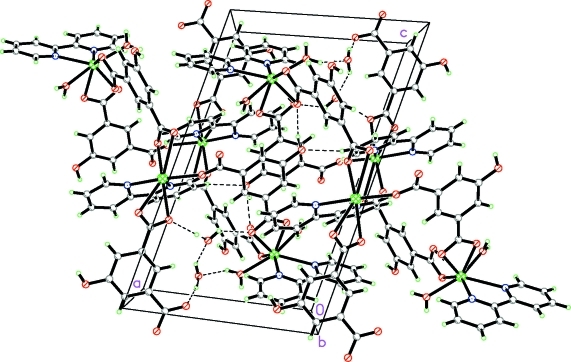
A view of the three-dimensional framework. Hydrogen bonds are shown as dashed lines.

## supplementary crystallographic information

### Comment

In recent years, *in situ* metal/ligand reactions have been widely investigated for the discovery of new organic reactions and the elucidation of reaction mechanism, as well as the generation of novel coordination polymers. There is currently much interest in employing polycarboxylate ligands with O donors and bipyridine ligands with N donors to design metal coordination polymers with intriguing structures and potential applications. Particular attention has been paid to 5-hydroxyisophthalate and 2,2'-bipyridine ligands; they have five O coordination sites and two N coordination sites, respectively, and thus can potentially afford different coordination modes in multicoordinated ways with transition metal ions (Li *et al.*, 2007; Plater *et al.*, 2001; Zhuo *et al.*, 2006*a*,b) to form new metal coordination polymers with various structures and interesting properties. In this paper, we report the crystal structure of the title compound, which was synthesized under hydrothermal conditions.

As shown in Fig. 1, both Dy^III^ ions exhibit a distorted tricapped trigonal prismatic geometry. One Dy^III^ ion is coordinated by five 5-hydroxyisophthalate (hip) ligands and one 2,2'-bipyridine (bpy) ligand and the other is by three hip ligands, one bpy ligand and two water molecules. The Dy—O bond lengths range from 2.287 (4) to 2.818 (4) Å and the Dy—N bond lengths range from 2.545 (5) to 2.628 (5) Å. In the crystal, the Dy^III^ ions are bridged by the carboxylate groups of the hip ligands, forming a three-dimensional framework (Fig. 2). O—H···O hydrogen bonds are present (Table 1).

### Experimental

A mixture of Dy_2_O_3_ (0.363 g, 1 mmol), 5-hydroxyisophthalic acid (0.182 g, 1 mmol), 2,2'-bipyridine (0.132 g, 1 mmol) and water (10 ml) in the presence of HClO_4_ (0.039 g, 0.385 mmol) was stirred vigorously for 30 min and then sealed in a 20 ml Teflon-lined stainless-steel autoclave. The autoclave was heated and maintained at 433 K for 50 h and then cooled to room temperature at 5 K h^-1^. Colorless block crystals were obtained.

### Refinement

H atoms of water molecules and hydroxyl groups were tentatively located in difference Fourier maps and refined as riding atoms, with O—H = 0.82 (hydroxyl) and 0.85 (water) Å and with *U*_iso_(H) = 1.5*U*_eq_(O). H atoms on C atoms were positioned geometrically and refined as riding atoms, with C—H = 0.93 Å and with *U*_iso_(H) = 1.2*U*_eq_(C). The highest residual electron density was found at 2.58 Å from H33 atom and the deepest hole at 0.51 Å from O4W atom.

### Crystal data

**Table:**

[Dy_2_(C_8_H_4_O_5_)_3_(C_10_H_8_N_2_)_2_(H_2_O)_2_]·2H_2_O	*Z* = 2
*M**_r_* = 1249.77	*F*(000) = 1224
Triclinic, *P*1	*D*_x_ = 1.829 Mg m^−^^3^
Hall symbol: -P 1	Mo *K*α radiation, λ = 0.71073 Å
*a* = 11.3736 (5) Å	Cell parameters from 7036 reflections
*b* = 12.0349 (6) Å	θ = 2.3–25.2°
*c* = 17.8360 (8) Å	µ = 3.35 mm^−^^1^
α = 91.310 (1)°	*T* = 298 K
β = 103.187 (1)°	Block, colourless
γ = 106.505 (1)°	0.25 × 0.24 × 0.21 mm
*V* = 2269.00 (18) Å^3^	

### Data collection

**Table:**

Bruker APEXII CCD diffractometer	8024 independent reflections
Radiation source: fine-focus sealed tube	7052 reflections with *I* > 2σ(*I*)
graphite	*R*_int_ = 0.020
φ and ω scans	θ_max_ = 25.2°, θ_min_ = 1.8°
Absorption correction: multi-scan (*SADABS*; Bruker, 2001)	*h* = −11→13
*T*_min_ = 0.488, *T*_max_ = 0.540	*k* = −14→12
11732 measured reflections	*l* = −21→14

### Refinement

**Table:**

Refinement on *F*^2^	Primary atom site location: structure-invariant direct methods
Least-squares matrix: full	Secondary atom site location: difference Fourier map
*R*[*F*^2^ > 2σ(*F*^2^)] = 0.035	Hydrogen site location: inferred from neighbouring sites
*wR*(*F*^2^) = 0.094	H-atom parameters constrained
*S* = 1.06	*w* = 1/[σ^2^(*F*_o_^2^) + (0.0438*P*)^2^ + 9.6294*P*] where *P* = (*F*_o_^2^ + 2*F*_c_^2^)/3
8024 reflections	(Δ/σ)_max_ = 0.001
624 parameters	Δρ_max_ = 2.65 e Å^−^^3^
252 restraints	Δρ_min_ = −1.25 e Å^−^^3^

### Fractional atomic coordinates and isotropic or equivalent isotropic displacement parameters (Å^2^)

**Table:**

	*x*	*y*	*z*	*U*_iso_*/*U*_eq_	
C1	0.4251 (5)	0.8899 (5)	0.7503 (3)	0.0251 (8)	
C2	0.3246 (5)	0.8628 (5)	0.6840 (3)	0.0239 (8)	
H2	0.3093	0.7969	0.6508	0.029*	
C3	0.2482 (5)	0.9350 (5)	0.6685 (3)	0.0244 (8)	
C4	0.2704 (5)	1.0328 (5)	0.7175 (3)	0.0293 (9)	
H4	0.2188	1.0809	0.7064	0.035*	
C5	0.3692 (6)	1.0599 (6)	0.7831 (4)	0.0322 (9)	
C6	0.4462 (6)	0.9871 (5)	0.7995 (4)	0.0302 (9)	
H6	0.5120	1.0043	0.8440	0.036*	
C7	0.5145 (5)	0.8182 (5)	0.7643 (3)	0.0238 (10)	
C8	0.1446 (5)	0.9091 (5)	0.5952 (3)	0.0234 (9)	
C9	0.0136 (5)	0.8915 (5)	0.1828 (3)	0.0245 (8)	
C10	0.1249 (5)	0.9454 (5)	0.1611 (3)	0.0249 (8)	
H10	0.1973	0.9860	0.1983	0.030*	
C11	0.1270 (5)	0.9381 (5)	0.0836 (3)	0.0249 (8)	
C12	0.0188 (5)	0.8775 (5)	0.0280 (3)	0.0249 (8)	
H12	0.0204	0.8723	−0.0238	0.030*	
C13	−0.0921 (5)	0.8246 (5)	0.0506 (3)	0.0259 (8)	
C14	−0.0937 (5)	0.8305 (5)	0.1277 (3)	0.0258 (8)	
H14	−0.1673	0.7932	0.1425	0.031*	
C15	0.0150 (5)	0.9011 (5)	0.2669 (3)	0.0241 (10)	
C16	−0.2103 (5)	0.7609 (5)	−0.0104 (3)	0.0274 (10)	
C17	0.5160 (5)	0.4159 (5)	0.6023 (3)	0.0211 (7)	
C18	0.3978 (5)	0.3324 (5)	0.5900 (3)	0.0212 (8)	
H18	0.3485	0.3307	0.6253	0.025*	
C19	0.3540 (5)	0.2526 (5)	0.5257 (3)	0.0201 (7)	
C20	0.4278 (5)	0.2559 (5)	0.4717 (3)	0.0216 (8)	
H20	0.4007	0.1998	0.4297	0.026*	
C21	0.5417 (5)	0.3438 (5)	0.4819 (3)	0.0222 (8)	
C22	0.5862 (5)	0.4218 (5)	0.5472 (3)	0.0229 (8)	
H22	0.6638	0.4788	0.5544	0.028*	
C23	0.2276 (5)	0.1624 (5)	0.5137 (3)	0.0203 (9)	
C24	0.5688 (5)	0.4978 (5)	0.6744 (3)	0.0220 (9)	
C25	1.0000 (8)	0.7669 (10)	0.8074 (5)	0.0683 (16)	
H25	0.9747	0.8291	0.7869	0.082*	
C26	1.1267 (8)	0.7756 (10)	0.8224 (6)	0.0719 (14)	
H26	1.1856	0.8417	0.8129	0.086*	
C27	1.1621 (9)	0.6827 (10)	0.8517 (6)	0.0729 (13)	
H27	1.2463	0.6840	0.8608	0.087*	
C28	1.0729 (8)	0.5862 (9)	0.8679 (5)	0.0685 (13)	
H28	1.0970	0.5233	0.8883	0.082*	
C29	0.9474 (8)	0.5858 (8)	0.8531 (5)	0.0610 (12)	
C30	0.8518 (8)	0.4909 (8)	0.8756 (5)	0.0602 (12)	
C31	0.8801 (9)	0.3964 (8)	0.9129 (5)	0.0648 (12)	
H31	0.9610	0.3886	0.9213	0.078*	
C32	0.7869 (9)	0.3161 (8)	0.9366 (6)	0.0685 (13)	
H32	0.8057	0.2552	0.9631	0.082*	
C33	0.6694 (9)	0.3243 (8)	0.9220 (5)	0.0684 (14)	
H33	0.6055	0.2697	0.9375	0.082*	
C34	0.6460 (9)	0.4171 (8)	0.8831 (5)	0.0661 (16)	
H34	0.5638	0.4221	0.8719	0.079*	
C35	0.3302 (6)	0.9129 (6)	0.3933 (4)	0.0425 (12)	
H35	0.3510	0.9844	0.4219	0.051*	
C36	0.4133 (7)	0.8951 (6)	0.3514 (4)	0.0439 (11)	
H36	0.4897	0.9515	0.3535	0.053*	
C37	0.3786 (7)	0.7913 (6)	0.3068 (4)	0.0416 (10)	
H37	0.4285	0.7779	0.2750	0.050*	
C38	0.2695 (6)	0.7073 (6)	0.3096 (4)	0.0380 (10)	
H38	0.2461	0.6358	0.2807	0.046*	
C39	0.1949 (6)	0.7292 (5)	0.3553 (4)	0.0339 (9)	
C40	0.0846 (6)	0.6358 (6)	0.3694 (4)	0.0356 (9)	
C41	0.0697 (7)	0.5191 (6)	0.3519 (4)	0.0409 (10)	
H41	0.1247	0.4972	0.3274	0.049*	
C42	−0.0277 (7)	0.4353 (6)	0.3710 (5)	0.0464 (11)	
H42	−0.0391	0.3565	0.3601	0.056*	
C43	−0.1078 (7)	0.4720 (6)	0.4069 (5)	0.0460 (11)	
H43	−0.1743	0.4182	0.4206	0.055*	
C44	−0.0871 (6)	0.5902 (6)	0.4219 (4)	0.0422 (12)	
H44	−0.1419	0.6141	0.4457	0.051*	
Dy1	0.04319 (2)	0.89445 (2)	0.429009 (13)	0.01643 (8)	
Dy2	0.67367 (2)	0.67220 (2)	0.800601 (14)	0.01969 (8)	
N1	0.9105 (5)	0.6742 (5)	0.8207 (3)	0.0390 (13)	
N2	0.7338 (6)	0.4996 (5)	0.8606 (3)	0.0407 (13)	
N3	0.0070 (4)	0.6719 (4)	0.4043 (3)	0.0238 (10)	
N4	0.2227 (5)	0.8338 (4)	0.3947 (3)	0.0298 (11)	
O1	0.4947 (4)	0.7273 (4)	0.7207 (2)	0.0282 (9)	
O2	0.6118 (4)	0.8495 (3)	0.8205 (2)	0.0267 (9)	
O3	0.0652 (4)	0.9668 (4)	0.5843 (3)	0.0312 (10)	
O4	0.1422 (4)	0.8342 (4)	0.5450 (2)	0.0324 (10)	
O5	0.3955 (6)	1.1574 (5)	0.8318 (3)	0.072 (2)	
H5	0.3461	1.1943	0.8150	0.108*	
O6	−0.0618 (4)	0.8244 (4)	0.2938 (2)	0.0256 (9)	
O7	0.0951 (4)	0.9848 (4)	0.3114 (2)	0.0307 (9)	
O8	−0.3077 (5)	0.7129 (6)	0.0117 (3)	0.0632 (18)	
O9	−0.2044 (4)	0.7600 (4)	−0.0800 (2)	0.0281 (9)	
O10	0.2343 (4)	0.9872 (4)	0.0599 (2)	0.0352 (10)	
H10A	0.2877	1.0299	0.0958	0.053*	
O11	0.6748 (4)	0.5741 (4)	0.6827 (2)	0.0324 (10)	
O12	0.5103 (4)	0.4909 (4)	0.7272 (2)	0.0283 (9)	
O13	0.1492 (3)	0.1884 (3)	0.5452 (2)	0.0263 (9)	
O14	0.2080 (3)	0.0689 (3)	0.4735 (2)	0.0247 (8)	
O15	0.6135 (4)	0.3572 (4)	0.4296 (2)	0.0368 (11)	
H15	0.5706	0.3213	0.3881	0.055*	
O1W	0.5317 (4)	0.6243 (4)	0.8801 (2)	0.0377 (11)	
H1WA	0.5604	0.6566	0.9261	0.057*	
H1WB	0.4515	0.6014	0.8712	0.057*	
O2W	0.7608 (4)	0.8059 (4)	0.7123 (2)	0.0337 (10)	
H2WA	0.7579	0.7565	0.6765	0.051*	
H2WB	0.7932	0.8733	0.6996	0.051*	
O3W	0.6892 (5)	0.5249 (5)	0.1047 (4)	0.0671 (17)	
H3WA	0.7242	0.5719	0.1452	0.101*	
H3WB	0.6780	0.5651	0.0666	0.101*	
O4W	0.2982 (7)	0.3417 (7)	0.7652 (4)	0.106 (3)	
H4WA	0.2287	0.2884	0.7480	0.160*	
H4WB	0.3365	0.3533	0.7291	0.160*	

### Atomic displacement parameters (Å^2^)

**Table:**

	*U*^11^	*U*^22^	*U*^33^	*U*^12^	*U*^13^	*U*^23^
C1	0.0205 (15)	0.0297 (16)	0.0218 (15)	0.0061 (13)	0.0008 (13)	−0.0021 (14)
C2	0.0201 (16)	0.0278 (17)	0.0217 (16)	0.0068 (14)	0.0016 (14)	−0.0009 (15)
C3	0.0198 (15)	0.0278 (16)	0.0231 (15)	0.0061 (13)	0.0017 (13)	−0.0006 (14)
C4	0.0235 (16)	0.0319 (17)	0.0287 (17)	0.0084 (15)	−0.0007 (15)	−0.0053 (15)
C5	0.0252 (17)	0.0354 (18)	0.0304 (18)	0.0085 (15)	−0.0025 (15)	−0.0083 (16)
C6	0.0234 (16)	0.0344 (18)	0.0274 (17)	0.0074 (15)	−0.0021 (15)	−0.0064 (16)
C7	0.0196 (19)	0.029 (2)	0.0202 (19)	0.0057 (17)	0.0013 (17)	−0.0010 (17)
C8	0.0201 (19)	0.026 (2)	0.021 (2)	0.0038 (17)	0.0030 (17)	0.0034 (17)
C9	0.0239 (15)	0.0292 (16)	0.0168 (15)	0.0044 (13)	0.0027 (13)	−0.0021 (14)
C10	0.0239 (16)	0.0295 (17)	0.0171 (16)	0.0040 (15)	0.0021 (14)	−0.0030 (15)
C11	0.0244 (17)	0.0298 (17)	0.0176 (16)	0.0050 (15)	0.0041 (14)	−0.0023 (15)
C12	0.0256 (16)	0.0295 (17)	0.0171 (16)	0.0057 (14)	0.0040 (14)	−0.0024 (15)
C13	0.0255 (15)	0.0303 (16)	0.0179 (15)	0.0043 (14)	0.0027 (13)	−0.0020 (14)
C14	0.0248 (16)	0.0306 (17)	0.0177 (16)	0.0034 (14)	0.0029 (14)	−0.0013 (15)
C15	0.0237 (19)	0.028 (2)	0.0172 (19)	0.0051 (17)	0.0024 (17)	−0.0017 (17)
C16	0.025 (2)	0.032 (2)	0.020 (2)	0.0041 (17)	0.0021 (17)	−0.0024 (18)
C17	0.0192 (14)	0.0225 (15)	0.0186 (15)	0.0031 (13)	0.0032 (13)	−0.0030 (13)
C18	0.0193 (15)	0.0234 (16)	0.0182 (16)	0.0024 (14)	0.0046 (13)	−0.0039 (14)
C19	0.0180 (14)	0.0230 (15)	0.0169 (14)	0.0032 (13)	0.0033 (12)	−0.0035 (13)
C20	0.0190 (15)	0.0245 (16)	0.0176 (16)	0.0024 (14)	0.0029 (14)	−0.0051 (14)
C21	0.0196 (16)	0.0256 (17)	0.0183 (16)	0.0028 (14)	0.0040 (14)	−0.0030 (14)
C22	0.0199 (16)	0.0253 (17)	0.0193 (16)	0.0013 (14)	0.0036 (14)	−0.0027 (14)
C23	0.0183 (18)	0.0230 (19)	0.0164 (18)	0.0027 (16)	0.0027 (16)	−0.0019 (17)
C24	0.0203 (19)	0.0223 (19)	0.0193 (19)	0.0034 (16)	0.0011 (16)	−0.0022 (17)
C25	0.043 (3)	0.104 (4)	0.063 (3)	0.024 (3)	0.019 (3)	0.021 (3)
C26	0.047 (2)	0.105 (3)	0.067 (3)	0.024 (2)	0.018 (2)	0.020 (3)
C27	0.050 (2)	0.102 (3)	0.070 (3)	0.028 (2)	0.015 (2)	0.017 (2)
C28	0.052 (2)	0.093 (3)	0.068 (3)	0.034 (2)	0.012 (2)	0.015 (2)
C29	0.052 (2)	0.080 (3)	0.061 (2)	0.038 (2)	0.011 (2)	0.010 (2)
C30	0.058 (2)	0.070 (3)	0.061 (2)	0.039 (2)	0.007 (2)	0.007 (2)
C31	0.066 (2)	0.065 (3)	0.067 (3)	0.035 (2)	0.002 (2)	0.008 (2)
C32	0.073 (3)	0.058 (3)	0.070 (3)	0.027 (2)	−0.001 (2)	0.009 (2)
C33	0.074 (3)	0.053 (3)	0.068 (3)	0.020 (2)	−0.003 (2)	0.011 (2)
C34	0.073 (3)	0.050 (3)	0.066 (3)	0.019 (3)	−0.003 (3)	0.010 (3)
C35	0.037 (2)	0.036 (2)	0.060 (3)	0.008 (2)	0.026 (2)	0.004 (2)
C36	0.039 (2)	0.039 (2)	0.058 (2)	0.0082 (18)	0.0248 (19)	0.0066 (19)
C37	0.039 (2)	0.038 (2)	0.054 (2)	0.0111 (17)	0.0248 (18)	0.0069 (18)
C38	0.0384 (19)	0.0341 (19)	0.048 (2)	0.0124 (16)	0.0220 (17)	0.0056 (17)
C39	0.0349 (19)	0.0294 (18)	0.044 (2)	0.0120 (16)	0.0201 (17)	0.0049 (17)
C40	0.0352 (19)	0.0289 (18)	0.047 (2)	0.0100 (16)	0.0192 (17)	0.0001 (17)
C41	0.0390 (19)	0.0305 (19)	0.056 (2)	0.0086 (16)	0.0196 (18)	−0.0044 (18)
C42	0.042 (2)	0.0324 (19)	0.064 (2)	0.0049 (17)	0.0189 (19)	−0.0042 (18)
C43	0.039 (2)	0.032 (2)	0.066 (2)	0.0034 (18)	0.020 (2)	−0.001 (2)
C44	0.035 (2)	0.030 (2)	0.064 (3)	0.005 (2)	0.021 (2)	−0.001 (2)
Dy1	0.01444 (13)	0.01919 (14)	0.01331 (14)	0.00240 (10)	0.00243 (10)	−0.00233 (10)
Dy2	0.01911 (14)	0.02153 (15)	0.01423 (14)	0.00364 (10)	−0.00064 (10)	−0.00393 (10)
N1	0.031 (3)	0.056 (4)	0.028 (3)	0.014 (3)	0.003 (2)	−0.008 (3)
N2	0.047 (3)	0.036 (3)	0.036 (3)	0.015 (3)	−0.002 (3)	0.002 (3)
N3	0.022 (2)	0.021 (2)	0.027 (3)	0.0041 (19)	0.007 (2)	−0.002 (2)
N4	0.025 (3)	0.028 (3)	0.039 (3)	0.007 (2)	0.015 (2)	0.001 (2)
O1	0.028 (2)	0.032 (2)	0.020 (2)	0.0113 (18)	−0.0043 (17)	−0.0067 (17)
O2	0.021 (2)	0.031 (2)	0.023 (2)	0.0085 (17)	−0.0051 (16)	−0.0102 (17)
O3	0.023 (2)	0.033 (2)	0.038 (2)	0.0123 (18)	0.0013 (18)	0.0082 (19)
O4	0.038 (2)	0.034 (2)	0.022 (2)	0.016 (2)	−0.0039 (18)	−0.0002 (18)
O5	0.074 (4)	0.069 (4)	0.063 (4)	0.047 (3)	−0.032 (3)	−0.047 (3)
O6	0.026 (2)	0.033 (2)	0.0132 (19)	0.0030 (17)	0.0032 (16)	0.0001 (16)
O7	0.035 (2)	0.032 (2)	0.015 (2)	−0.0036 (18)	0.0041 (17)	−0.0036 (17)
O8	0.033 (3)	0.103 (5)	0.024 (3)	−0.023 (3)	0.005 (2)	−0.006 (3)
O9	0.028 (2)	0.036 (2)	0.0140 (19)	0.0053 (18)	−0.0009 (16)	−0.0052 (17)
O10	0.027 (2)	0.045 (3)	0.027 (2)	−0.0024 (19)	0.0107 (19)	−0.009 (2)
O11	0.028 (2)	0.031 (2)	0.028 (2)	−0.0036 (18)	0.0046 (18)	−0.0111 (18)
O12	0.030 (2)	0.029 (2)	0.022 (2)	0.0026 (17)	0.0078 (17)	−0.0076 (17)
O13	0.0190 (19)	0.027 (2)	0.030 (2)	0.0000 (16)	0.0095 (17)	−0.0094 (17)
O14	0.0198 (19)	0.023 (2)	0.026 (2)	−0.0008 (16)	0.0057 (16)	−0.0103 (17)
O15	0.024 (2)	0.054 (3)	0.024 (2)	−0.004 (2)	0.0114 (18)	−0.012 (2)
O1W	0.027 (2)	0.049 (3)	0.025 (2)	−0.006 (2)	0.0046 (18)	−0.008 (2)
O2W	0.042 (3)	0.027 (2)	0.028 (2)	0.0007 (19)	0.011 (2)	0.0007 (18)
O3W	0.062 (4)	0.058 (4)	0.064 (4)	−0.007 (3)	0.014 (3)	−0.003 (3)
O4W	0.095 (5)	0.122 (7)	0.056 (4)	−0.047 (5)	0.030 (4)	−0.019 (4)

### Geometric parameters (Å, °)

**Table:**

C1—C6	1.377 (8)	C30—C31	1.409 (12)
C1—C2	1.401 (8)	C31—C32	1.371 (13)
C1—C7	1.494 (8)	C31—H31	0.9300
C2—C3	1.383 (8)	C32—C33	1.334 (13)
C2—H2	0.9300	C32—H32	0.9300
C3—C4	1.377 (8)	C33—C34	1.385 (12)
C3—C8	1.505 (8)	C33—H33	0.9300
C4—C5	1.383 (8)	C34—N2	1.336 (11)
C4—H4	0.9300	C34—H34	0.9300
C5—O5	1.362 (8)	C35—N4	1.325 (8)
C5—C6	1.397 (9)	C35—C36	1.389 (9)
C6—H6	0.9300	C35—H35	0.9300
C7—O1	1.263 (7)	C36—C37	1.371 (10)
C7—O2	1.269 (7)	C36—H36	0.9300
C8—O4	1.246 (7)	C37—C38	1.372 (9)
C8—O3	1.272 (7)	C37—H37	0.9300
C9—C14	1.381 (8)	C38—C39	1.372 (9)
C9—C10	1.393 (8)	C38—H38	0.9300
C9—C15	1.499 (7)	C39—N4	1.345 (8)
C10—C11	1.390 (8)	C39—C40	1.502 (9)
C10—H10	0.9300	C40—N3	1.346 (7)
C11—O10	1.362 (7)	C40—C41	1.386 (9)
C11—C12	1.388 (8)	C41—C42	1.384 (10)
C12—C13	1.396 (8)	C41—H41	0.9300
C12—H12	0.9300	C42—C43	1.383 (10)
C13—C14	1.380 (8)	C42—H42	0.9300
C13—C16	1.511 (8)	C43—C44	1.384 (9)
C14—H14	0.9300	C43—H43	0.9300
C15—O7	1.260 (7)	C44—N3	1.335 (8)
C15—O6	1.265 (7)	C44—H44	0.9300
C16—O8	1.249 (7)	Dy1—O13^i^	2.287 (4)
C16—O9	1.260 (7)	Dy1—O3^ii^	2.328 (4)
C17—C22	1.392 (7)	Dy1—O4	2.355 (4)
C17—C18	1.397 (7)	Dy1—O14^iii^	2.358 (4)
C17—C24	1.497 (8)	Dy1—O6	2.444 (4)
C18—C19	1.380 (7)	Dy1—O7	2.496 (4)
C18—H18	0.9300	Dy1—N4	2.545 (5)
C19—C20	1.409 (7)	Dy1—N3	2.603 (5)
C19—C23	1.502 (7)	Dy1—O3	2.818 (4)
C20—C21	1.391 (7)	Dy2—O9^iv^	2.300 (4)
C20—H20	0.9300	Dy2—O1W	2.346 (4)
C21—O15	1.359 (7)	Dy2—O11	2.393 (4)
C21—C22	1.380 (8)	Dy2—O2W	2.444 (4)
C22—H22	0.9300	Dy2—O1	2.470 (4)
C23—O14	1.257 (6)	Dy2—O2	2.472 (4)
C23—O13	1.260 (6)	Dy2—O12	2.527 (4)
C24—O12	1.262 (7)	Dy2—N2	2.549 (6)
C24—O11	1.266 (7)	Dy2—N1	2.628 (5)
C25—N1	1.346 (11)	O5—H5	0.8200
C25—C26	1.377 (12)	O10—H10A	0.8200
C25—H25	0.9300	O15—H15	0.8200
C26—C27	1.367 (14)	O1W—H1WA	0.8500
C26—H26	0.9300	O1W—H1WB	0.8500
C27—C28	1.394 (14)	O2W—H2WA	0.8501
C27—H27	0.9300	O2W—H2WB	0.8500
C28—C29	1.390 (11)	O3W—H3WA	0.8500
C28—H28	0.9300	O3W—H3WB	0.8501
C29—N1	1.347 (10)	O4W—H4WA	0.8500
C29—C30	1.470 (13)	O4W—H4WB	0.8500
C30—N2	1.343 (10)		
			
C6—C1—C2	119.8 (5)	C42—C41—C40	119.5 (6)
C6—C1—C7	120.1 (5)	C42—C41—H41	120.2
C2—C1—C7	120.0 (5)	C40—C41—H41	120.2
C3—C2—C1	119.5 (5)	C43—C42—C41	118.2 (7)
C3—C2—H2	120.2	C43—C42—H42	120.9
C1—C2—H2	120.2	C41—C42—H42	120.9
C4—C3—C2	120.5 (5)	C42—C43—C44	118.8 (7)
C4—C3—C8	120.1 (5)	C42—C43—H43	120.6
C2—C3—C8	119.3 (5)	C44—C43—H43	120.6
C3—C4—C5	120.4 (6)	N3—C44—C43	123.6 (6)
C3—C4—H4	119.8	N3—C44—H44	118.2
C5—C4—H4	119.8	C43—C44—H44	118.2
O5—C5—C4	121.8 (6)	O13^i^—Dy1—O3^ii^	72.52 (14)
O5—C5—C6	118.7 (5)	O13^i^—Dy1—O4	89.48 (15)
C4—C5—C6	119.5 (6)	O3^ii^—Dy1—O4	127.26 (15)
C1—C6—C5	120.3 (6)	O13^i^—Dy1—O14^iii^	134.77 (13)
C1—C6—H6	119.8	O3^ii^—Dy1—O14^iii^	77.83 (14)
C5—C6—H6	119.8	O4—Dy1—O14^iii^	82.03 (15)
O1—C7—O2	119.7 (5)	O13^i^—Dy1—O6	86.34 (13)
O1—C7—C1	120.7 (5)	O3^ii^—Dy1—O6	87.44 (14)
O2—C7—C1	119.6 (5)	O4—Dy1—O6	141.71 (14)
O4—C8—O3	121.5 (5)	O14^iii^—Dy1—O6	125.92 (13)
O4—C8—C3	118.6 (5)	O13^i^—Dy1—O7	129.91 (14)
O3—C8—C3	119.8 (5)	O3^ii^—Dy1—O7	77.91 (15)
C14—C9—C10	120.3 (5)	O4—Dy1—O7	140.19 (15)
C14—C9—C15	121.7 (5)	O14^iii^—Dy1—O7	73.56 (13)
C10—C9—C15	118.1 (5)	O6—Dy1—O7	52.45 (13)
C11—C10—C9	119.6 (5)	O13^i^—Dy1—N4	139.44 (15)
C11—C10—H10	120.2	O3^ii^—Dy1—N4	145.09 (16)
C9—C10—H10	120.2	O4—Dy1—N4	76.16 (16)
O10—C11—C12	118.2 (5)	O14^iii^—Dy1—N4	81.22 (15)
O10—C11—C10	121.6 (5)	O6—Dy1—N4	82.61 (15)
C12—C11—C10	120.3 (5)	O7—Dy1—N4	69.50 (16)
C11—C12—C13	119.5 (5)	O13^i^—Dy1—N3	76.12 (14)
C11—C12—H12	120.2	O3^ii^—Dy1—N3	141.60 (15)
C13—C12—H12	120.2	O4—Dy1—N3	73.24 (15)
C14—C13—C12	120.2 (5)	O14^iii^—Dy1—N3	140.54 (14)
C14—C13—C16	120.5 (5)	O6—Dy1—N3	68.80 (14)
C12—C13—C16	119.3 (5)	O7—Dy1—N3	107.11 (14)
C13—C14—C9	120.2 (5)	N4—Dy1—N3	63.48 (15)
C13—C14—H14	119.9	O13^i^—Dy1—O3	73.04 (13)
C9—C14—H14	119.9	O3^ii^—Dy1—O3	78.01 (14)
O7—C15—O6	119.7 (5)	O4—Dy1—O3	49.26 (13)
O7—C15—C9	119.8 (5)	O14^iii^—Dy1—O3	67.89 (13)
O6—C15—C9	120.4 (5)	O6—Dy1—O3	157.51 (12)
O8—C16—O9	124.3 (5)	O7—Dy1—O3	137.87 (13)
O8—C16—C13	117.8 (5)	N4—Dy1—O3	118.94 (14)
O9—C16—C13	118.0 (5)	N3—Dy1—O3	113.26 (13)
C22—C17—C18	119.5 (5)	O9^iv^—Dy2—O1W	76.99 (14)
C22—C17—C24	119.6 (5)	O9^iv^—Dy2—O11	144.48 (14)
C18—C17—C24	120.9 (5)	O1W—Dy2—O11	130.31 (14)
C19—C18—C17	120.2 (5)	O9^iv^—Dy2—O2W	102.70 (15)
C19—C18—H18	119.9	O1W—Dy2—O2W	147.49 (16)
C17—C18—H18	119.9	O11—Dy2—O2W	67.17 (14)
C18—C19—C20	120.1 (5)	O9^iv^—Dy2—O1	126.34 (14)
C18—C19—C23	119.6 (5)	O1W—Dy2—O1	80.60 (15)
C20—C19—C23	120.3 (5)	O11—Dy2—O1	84.89 (14)
C21—C20—C19	119.3 (5)	O2W—Dy2—O1	73.59 (14)
C21—C20—H20	120.4	O9^iv^—Dy2—O2	74.47 (14)
C19—C20—H20	120.4	O1W—Dy2—O2	74.31 (15)
O15—C21—C22	117.2 (5)	O11—Dy2—O2	129.29 (14)
O15—C21—C20	122.6 (5)	O2W—Dy2—O2	74.37 (14)
C22—C21—C20	120.3 (5)	O1—Dy2—O2	52.60 (12)
C21—C22—C17	120.5 (5)	O9^iv^—Dy2—O12	145.62 (14)
C21—C22—H22	119.8	O1W—Dy2—O12	77.65 (14)
C17—C22—H22	119.8	O11—Dy2—O12	52.71 (13)
O14—C23—O13	125.5 (5)	O2W—Dy2—O12	111.16 (13)
O14—C23—C19	118.4 (5)	O1—Dy2—O12	71.06 (14)
O13—C23—C19	116.1 (5)	O2—Dy2—O12	119.80 (13)
O12—C24—O11	119.8 (5)	O9^iv^—Dy2—N2	79.69 (17)
O12—C24—C17	121.1 (5)	O1W—Dy2—N2	80.15 (18)
O11—C24—C17	119.1 (5)	O11—Dy2—N2	83.40 (17)
N1—C25—C26	124.2 (10)	O2W—Dy2—N2	132.21 (18)
N1—C25—H25	117.9	O1—Dy2—N2	142.38 (17)
C26—C25—H25	117.9	O2—Dy2—N2	146.97 (16)
C27—C26—C25	117.1 (10)	O12—Dy2—N2	73.40 (16)
C27—C26—H26	121.5	O9^iv^—Dy2—N1	67.91 (15)
C25—C26—H26	121.5	O1W—Dy2—N1	132.17 (17)
C26—C27—C28	120.5 (9)	O11—Dy2—N1	76.59 (15)
C26—C27—H27	119.7	O2W—Dy2—N1	73.89 (17)
C28—C27—H27	119.7	O1—Dy2—N1	146.81 (16)
C29—C28—C27	118.7 (9)	O2—Dy2—N1	122.85 (16)
C29—C28—H28	120.6	O12—Dy2—N1	115.59 (16)
C27—C28—H28	120.6	N2—Dy2—N1	62.81 (19)
N1—C29—C28	121.2 (9)	C25—N1—C29	118.1 (7)
N1—C29—C30	118.0 (7)	C25—N1—Dy2	122.0 (5)
C28—C29—C30	120.8 (8)	C29—N1—Dy2	119.5 (5)
N2—C30—C31	120.4 (9)	C34—N2—C30	117.7 (7)
N2—C30—C29	116.4 (7)	C34—N2—Dy2	118.8 (5)
C31—C30—C29	123.1 (8)	C30—N2—Dy2	123.3 (5)
C32—C31—C30	119.2 (9)	C44—N3—C40	117.4 (5)
C32—C31—H31	120.4	C44—N3—Dy1	123.9 (4)
C30—C31—H31	120.4	C40—N3—Dy1	118.7 (4)
C33—C32—C31	120.7 (9)	C35—N4—C39	118.0 (5)
C33—C32—H32	119.7	C35—N4—Dy1	120.5 (4)
C31—C32—H32	119.7	C39—N4—Dy1	118.3 (4)
C32—C33—C34	117.7 (10)	C7—O1—Dy2	93.8 (3)
C32—C33—H33	121.1	C7—O2—Dy2	93.6 (3)
C34—C33—H33	121.1	C8—O3—Dy1^ii^	168.2 (4)
N2—C34—C33	124.2 (9)	C8—O3—Dy1	81.1 (3)
N2—C34—H34	117.9	Dy1^ii^—O3—Dy1	101.99 (14)
C33—C34—H34	117.9	C8—O4—Dy1	103.3 (3)
N4—C35—C36	123.6 (7)	C5—O5—H5	109.5
N4—C35—H35	118.2	C15—O6—Dy1	94.6 (3)
C36—C35—H35	118.2	C15—O7—Dy1	92.3 (3)
C37—C36—C35	117.5 (6)	C16—O9—Dy2^v^	139.1 (4)
C37—C36—H36	121.3	C11—O10—H10A	109.5
C35—C36—H36	121.3	C24—O11—Dy2	96.7 (3)
C36—C37—C38	119.3 (6)	C24—O12—Dy2	90.5 (3)
C36—C37—H37	120.4	C23—O13—Dy1^i^	140.5 (4)
C38—C37—H37	120.4	C23—O14—Dy1^vi^	138.4 (3)
C39—C38—C37	119.8 (7)	C21—O15—H15	109.5
C39—C38—H38	120.1	Dy2—O1W—H1WA	115.0
C37—C38—H38	120.1	Dy2—O1W—H1WB	133.5
N4—C39—C38	121.5 (6)	H1WA—O1W—H1WB	107.7
N4—C39—C40	116.2 (5)	Dy2—O2W—H2WA	99.2
C38—C39—C40	122.2 (6)	Dy2—O2W—H2WB	153.0
N3—C40—C41	122.5 (6)	H2WA—O2W—H2WB	107.7
N3—C40—C39	116.2 (5)	H3WA—O3W—H3WB	107.7
C41—C40—C39	121.2 (6)	H4WA—O4W—H4WB	107.7

Symmetry codes: (i) −*x*, −*y*+1, −*z*+1; (ii) −*x*, −*y*+2, −*z*+1; (iii) *x*, *y*+1, *z*; (iv) *x*+1, *y*, *z*+1; (v) *x*−1, *y*, *z*−1; (vi) *x*, *y*−1, *z*.

### Hydrogen-bond geometry (Å, °)

**Table:**

*D*—H···*A*	*D*—H	H···*A*	*D*···*A*	*D*—H···*A*
O5—H5···O4W^iii^	0.82	2.15	2.910 (11)	154
O10—H10A···O2^vii^	0.82	1.95	2.761 (6)	168
O15—H15···O1^viii^	0.82	1.92	2.718 (6)	163
O1W—H1WA···O8^iv^	0.85	1.84	2.609 (6)	150
O1W—H1WB···O3W^viii^	0.85	2.01	2.717 (7)	141
O2W—H2WA···O11	0.85	2.15	2.676 (6)	120
O2W—H2WB···O7^vii^	0.85	1.86	2.689 (6)	164
O3W—H3WA···O4W^viii^	0.85	1.99	2.744 (9)	147
O3W—H3WB···O8^ix^	0.85	2.03	2.832 (9)	156
O4W—H4WA···O6^i^	0.85	1.96	2.809 (8)	175
O4W—H4WB···O12	0.85	2.20	2.801 (8)	128

Symmetry codes: (iii) *x*, *y*+1, *z*; (vii) −*x*+1, −*y*+2, −*z*+1; (viii) −*x*+1, −*y*+1, −*z*+1; (iv) *x*+1, *y*, *z*+1; (ix) *x*+1, *y*, *z*; (i) −*x*, −*y*+1, −*z*+1.
